# HHOMR: a hybrid high-order moment residual model for miRNA-disease association prediction

**DOI:** 10.1093/bib/bbae412

**Published:** 2024-08-23

**Authors:** Zhengwei Li, Lipeng Wan, Lei Wang, Wenjing Wang, Ru Nie

**Affiliations:** School of Computer Science and Technology, China University of Mining and Technology, Xuzhou 221116, China; Guangxi Academy of Science, Nanning, 530007, China; School of Information Science and Engineering, Zaozhuang University, Zaozhuang, 277160, China; School of Computer Science and Technology, China University of Mining and Technology, Xuzhou 221116, China; School of Computer Science and Technology, China University of Mining and Technology, Xuzhou 221116, China; Guangxi Academy of Science, Nanning, 530007, China; School of Computer Science and Technology, China University of Mining and Technology, Xuzhou 221116, China; School of Computer Science and Technology, China University of Mining and Technology, Xuzhou 221116, China; Mine Digitization Engineering Research Center of the Ministry of Education, China University of Mining and Technology, Xuzhou 221116, China

**Keywords:** higher-order moments, graph neural networks, miRNA–disease associations, attention mechanisms

## Abstract

Numerous studies have demonstrated that microRNAs (miRNAs) are critically important for the prediction, diagnosis, and characterization of diseases. However, identifying miRNA–disease associations through traditional biological experiments is both costly and time-consuming. To further explore these associations, we proposed a model based on hybrid high-order moments combined with element-level attention mechanisms (HHOMR). This model innovatively fused hybrid higher-order statistical information along with structural and community information. Specifically, we first constructed a heterogeneous graph based on existing associations between miRNAs and diseases. HHOMR employs a structural fusion layer to capture structure-level embeddings and leverages a hybrid high-order moments encoder layer to enhance features. Element-level attention mechanisms are then used to adaptively integrate the features of these hybrid moments. Finally, a multi-layer perceptron is utilized to calculate the association scores between miRNAs and diseases. Through five-fold cross-validation on HMDD v2.0, we achieved a mean AUC of 93.28%. Compared with four state-of-the-art models, HHOMR exhibited superior performance. Additionally, case studies on three diseases—esophageal neoplasms, lymphoma, and prostate neoplasms—were conducted. Among the top 50 miRNAs with high disease association scores, 46, 47, and 45 associated with these diseases were confirmed by the dbDEMC and miR2Disease databases, respectively. Our results demonstrate that HHOMR not only outperforms existing models but also shows significant potential in predicting miRNA–disease associations.

## Introduction

MicroRNA (miRNA) is an endogenous, single-stranded, non-coding RNA molecule, typically 19–25 nucleotides in length [1]. Numerous studies have linked the onset of diseases to anomalous miRNA expression, highlighting its crucial role in the pathogenesis of various conditions [2–4]. For example, human breast cancer tissues show abnormal miRNA expression levels, such as miR-155, miR-21, miR-145, and miR- 125b, compared to normal breast tissues [5]. Consequently, identifying associations between miRNAs and diseases (MDAs) proves greatly beneficial for disease prevention and treatment.

Researchers from diverse fields, including immunology, genetics, bioinformatics, and cell biology, have invested significant efforts in exploring potential connections between miRNAs and diseases [6]. Initially, these investigations primarily relied on wet laboratory techniques such as microarray analysis [7], primer extension [8], and Polymerase Chain Reaction [9]. Although these methods provided high prediction accuracy, they were time-intensive and costly. However, with rapid advancements in computer technology and increased computational capabilities of hardware, the focus has shifted toward computational methods. Building on the foundation of earlier research, miRNA–disease datasets such as miR2Disease [10] and HMDDv2.0 [11] have equipped computational approaches with robust and accurate data support. These methods, supported by extensive experimental validation, have proven effective in predicting miRNA–disease associations, offering substantial time and cost savings. The fundamental premise of early computational methods posits that miRNAs with similar functions are often linked to diseases with analogous phenotypic characteristics, and vice versa. For instance, Mork *et al.* [12] used associations between miRNAs and proteins, along with protein-disease connections, to predict miRNA–disease associations, with proteins serving as intermediaries. Gao *et al.* [13, 14] utilized support vector machine (SVM) technology to analyze six biologically meaningful sequence features, assessing the coding potential of transcripts quickly and accurately. Zuo *et al.* [15] implemented a ranking-based model using K-Nearest Neighbors (KNN) to explore potential MDAs. This model employs KNN to identify miRNAs and diseases, and then uses a Support Vector Machine ranking model to reorder the KNN results, guided by a weighted voting process to definitively rank miRNA–disease pairs. Jiang *et al.* [16] introduced a computational method utilizing hypergeometric probability distributions to investigate disease-related miRNAs. Additionally, Luo *et al.* [17] developed a method based on transductive learning, systematically ordering miRNAs in relation to diseases by integrating similarity measures with known miRNA–disease associations. Recently, the field of deep learning has seen rapid advancements. Many researchers are integrating it into their study areas, including the prediction of miRNA–disease relationships. Ji *et al.* [18] introduced the Autoencoder-based MiRNA-Disease Association (AEMDA) computational framework, which features a learning-based method for extracting features, thereby aiding in the construction of miRNA and disease feature representations. The use of deep autoencoders and the reconstruction error method enables the prediction of MDAs. Xu *et al.* [19] applied the Probabilistic Matrix Factorization algorithm, commonly utilized in recommender systems, to effectively leverage all available data in recommending miRNAs associated with diseases. Similarly, Liu *et al.* [20] proposed the Deep Forest-based Embedding Learning for MiRNA-Disease Association computational approach, involving two deep autoencoders for low-dimensional feature representations and a deep random forest to obtain prediction scores for unlabeled MDAs. In efforts to prioritize the detection of MDAs without relying on non-positive samples, Chen *et al.* [21] proposed a semi-supervised classification approach that facilitates the prediction of associations for isolated diseases. Likewise, the Stacked AEMDA [22] involved pre-training Stacked Autoencoders with all MDA pairs, followed by fine-tuning with an equivalent amount of observed and unknown MDA pairs to identify potential disease-related miRNAs.

In recent years, the graph neural network has emerged as a notably prominent network model, gaining widespread attention for its ability to abstract complex relationships into simpler graph structures, particularly excelling in fields such as bioinformatics. For example, Yu *et al.* [23] introduced the Layer-wise Attention Graph Convolutional Network (GCN), which integrates drug–disease associations along with drug–drug and disease–disease similarities into a unified heterogeneous graph. This model employs graph convolutional operations to generate node embeddings, demonstrating its efficacy in predicting associations. Additionally, Yu *et al.* [24] developed a meta-path-based method that defines seven symmetric meta-paths based on various semantic criteria within the miRNA–disease–gene heterogeneous information network. This method updates node features according to these meta-paths and uses the resulting features to compute final association scores. Tang *et al.* [25] proposed the Multi-modal GCN model, which combines GCN encoders and Graph Attention Network encoders to extract miRNA and disease features, further enhanced by multi-channel attention mechanisms.

**Figure 1:**
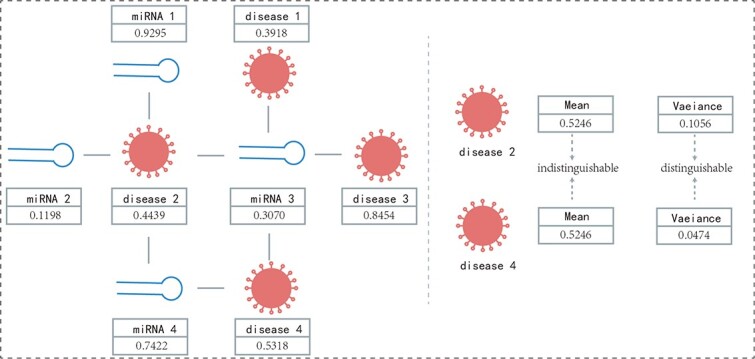
Heterogeneous graph of miRNAs and disease. The left side depicts a heterogeneous graph of miRNA–disease associations, displaying eigenvalues as the values of the nodes. The right side illustrates that the first-order moments (means) of the nodes for disease 2 and disease 4 are unable to distinguish between these two nodes, whereas the second-order moments (variances) provide clear differentiation.

Despite the demonstrated effectiveness of these methods, they are not without issues. For graph-structured models, the prevalent use of a single statistical measure to aggregate neighborhood information, such as the mean or maximum, often results in a significant loss of statistical information from the nodes. To illustrate the importance of employing more comprehensive statistics, consider the heterogeneous graph of miRNAs and diseases shown in Fig. 1. It is constructed from validated associations between miRNAs and diseases, with edges connecting nodes if an association exists. The eigenvalues of the nodes are calculated based on semantic similarity of diseases, functional similarity of miRNAs, and similarity based on the Gaussian interaction profile kernel. Subsequently, employing a single statistic to aggregate neighborhood features results in identical features for both disease2 and disease4 at 0.5246, making it impossible to distinguish accurately between the two nodes, which adversely affects the evaluation of subsequent associations. If higher-order statistics, such as variance, are used, the distinctions become clearer, with variances of 0.1056 and 0.0474 for disease2 and disease4, respectively, thus significantly enhancing the model’s performance.

In this study, we introduce a model named HHOMR, which utilizes high-order moment features for node feature aggregation. Initially, we constructed a heterogeneous graph based on verified MDAs and a feature matrix for two types of nodes, derived from their similarity relationships. We then integrated spatial and feature domain information as inputs to the model, while high-dimensional distribution information was processed through a hybrid high-order moment module. Subsequently, an element-based attention mechanism was introduced, allowing the model to adaptively learn the nodes’ higher-order moment information. A residual structure was employed to merge the node features from the previous layer with the processed information, thereby enhancing the model’s representational capabilities. The final step involved using a fully connected layer to generate scores related to miRNAs and diseases. To validate the model’s effectiveness, we employed a five-fold cross-validation method in our experiments, achieving an AUC of 93.28%, which indicates favorable results. Moreover, when compared to four advanced models—MPM, MuCoMiD, NEMII, and VGAMF—our model consistently exhibited superior performance. Additionally, to better demonstrate the model’s practical utility in predicting potential disease–miRNA associations, we conducted case studies on three diseases: prostate cancer, esophageal cancer, and lymphoma. We evaluated the predicted associations using the miR2Disease and dbDEMC datasets, finding that 46, 47, and 45 of the top 50 miRNAs were validated for these diseases, respectively. These results underscore the potential of the HHOMR as a valuable tool for researchers exploring MDAs.

## Materials and dataset

### Datasets

The datasets utilized in this study comprises 5430 validated pairs of miRNA–disease associations, sourced from the publicly available datasets HMDD2.0. It can be found at *https://www.cuilab.cn/hmdd* [11]. Within this datasets, there are a total of $q_{d}\left ( 383\right )$ distinct diseases and $q_{m}\left ( 495\right )$ miRNAs. Based on the aforementioned datasets, a correlation matrix $ \mathcal{R} $ was constructed for MDAs. The dimension of the matrix is $q_{d} \times q_{m}$, and the element value is set to 1 if miRNA is associated with disease, and 0 otherwise. It is important to note that $ \mathcal{R} $ of 0 does not necessarily indicate a lack of association, rather it denotes that the relationship between the miRNA and the disease is unknown. The matrix $ \mathcal{R} $ is defined as follows: 


(1) \begin{align*}& \mathcal{R}\left ( i,j\right )=\left\{\begin{matrix} 1 & miRNA\ i\ and \ disease\ j \ has \ relationship \\ 0 & otherwise \end{matrix}\right.\end{align*}

### Semantic similarity of diseases

**Figure 2:**
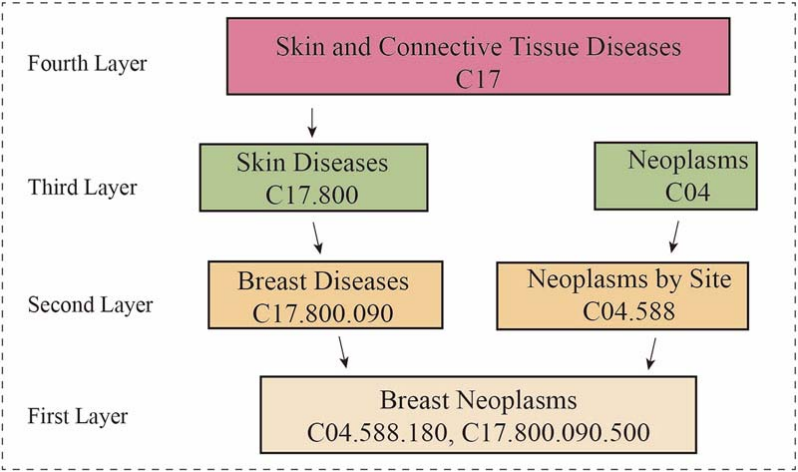
DAG of breast neoplasms. Each block within the diagram includes the category of the disease along with the MeSH Unique ID, which is used to uniquely identify a class of diseases. The semantic information of the root node, ’Breast Neoplasms’, is derived by weighting and summing the values from the ancestor nodes.

The US National Library of Medicine describes the terms of diseases in a detailed hierarchy, with specific data from *http://www.nlm.nih.gov/*. A study [26] has built on this to construct these terms into a directed acyclic graph to represent the semantic information of diseases. Thus we constructed a layered directed acyclic graph ($DAG$) for each disease utilizing the hierarchical tree structure provided by MeSH. Figure 2 shows the $DAG$ of a breast tumor. Here, a disease can be represented as $DAG(d_{i}) = (d_{i}, T_{d_{i}}, E_{d_{i}})$, where $d_{i}$ denotes the disease, $E_{d_{i}}$ represents the set of directly adjacent edges between nodes, and $T_{d_{i}}$ represents all ancestor nodes of the disease. Each disease in the $DAG$ receives semantic information from its ancestor nodes, demonstrated as follows: 


(2) \begin{align*}& \mathcal{D}_{d_{m}}\left ( d_{n} \right )=\left\{\begin{matrix} 1 & d_{n}=d_{m} \\ max\left \{ \delta * \mathcal{D}_{d_{m}}\left ( {d_{n}}^{\prime} \right ) \mid{d_{n}}^{\prime}\in \;children\;\, of\;\,d_{n} \right \} & d_{n}\neq d_{m} \end{matrix}\right.\end{align*}

The contribution value diminishes with the increasing distance between the disease node and its ancestor node; $\delta $ represents the extent of semantic attenuation. According to the research conducted by Chen *et al.* [27], it has been found that an attenuation value of 0.5 yields the most effective semantic expression. Therefore, in this context, $\delta $ is set to 0.5. If there are more shared nodes between the DAG graphs of diseases, then the semantic similarity between the diseases is greater. Based on the aforementioned hypothesis, the semantic similarity between all diseases can be computed as follows: 


(3) \begin{align*}& \mathcal{MDSS}(d_{i}, d_{j})=\frac{\sum_{ d_{k}\in T_{ d_{i}} \bigcap T_{ d_{j}} } ( D_{d_{i}}(d_{k})+ D_{d_{j}}(d_{k})) }{\sum_{d_{k}\in T_{d_{i}}} D_{d_{i}}(d_{k}) + \sum_{d_{k}\in T_{d_{j}}} D_{d_{j}}(d_{k})}\end{align*}

### Functional similarity of miRNAs

Several studies [28, 29] have substantiated the intricate associations between miRNAs and diseases, leading to an increased research focus on investigating the specific nature of miRNA–disease relationships. Research findings have indicated that when two miRNAs exhibit functional similarity, they are highly likely to be strongly associated with phenotypically similar diseases [30]. Conversely, if there is a substantial functional disparity between two miRNAs, the diseases they are associated with will manifest markedly distinct characteristics. Building upon these insights, Wang *et al.* [28] devised an algorithm to accurately determine the functional relevance between miRNAs. Consequently, we obtained miRNA similarity score data from *https://www.cuilab.cn/files/images/cuilab/misim.zip* and processed it to construct a $495\times 495$ adjacency matrix. Each element of this matrix represents the similarity score between the $i$th and $j$th miRNAs. In particular, for two miRNAs, denoted as $ \mathcal{M R}_{1}$ and $ \mathcal{M R}_{2}$, let $\mathcal{D T}_{1} = \left \{ r_{1 1},r_{1 2},...,r_{1 m} \right \}$ and $\mathcal{D T}_{2} = \left \{ r_{2 1}, r_{2 2},...,r_{2 n} \right \}$ represent the sets of diseases associated with $ \mathcal{M R}_{1}$ and $ \mathcal{M R}_{2}$, respectively. The functional similarity between two miRNAs is delineated as follows: 


(4) \begin{align*}& \mathcal{F M R S}\left(\mathcal{M R}_{1}, \mathcal{M R}_{2}\right)=\frac{\sum_{i=1}^{m} Sim \left(r_{1 i}, \mathcal{D T}_{2}\right)+\sum_{j=1}^{n} Sim \left(r_{2 j}, \mathcal{D T}_{1}\right)}{m+n}\end{align*}

where $m$ and $n$, respectively, denote the number of diseases in the sets $\mathcal{D T}_{1}$ and $\mathcal{D T}_{2}$. $Sim(r,\mathcal{D}\mathcal{T})$ is the maximum similarity between a disease and a disease group. It is calculated as follows: 


(5) \begin{align*}& Sim(r,\mathcal{D}\mathcal{T}) = {MAX}_{{r}_{i}\in \mathcal{DT}}(\mathcal{MDSS}(r,{r}_{i}))\end{align*}

### Similarity based on the Gaussian interaction profile kernel

To acquire more detailed similarity information and address missing data regarding similarities between diseases and miRNAs, this section introduces the Gaussian Interaction Profile Kernel Similarity [31]. Research conducted by Lu *et al.* [30] has demonstrated that miRNAs highly correlated with similar diseases exhibit greater functional similarity, and vice versa. In this context, $m_{i}$ denotes the $i$th miRNA, binary vectors $BV(m_{i})$ are employed to denote the presence or absence of associations between miRNAs and known diseases. Subsequently, we can calculate the Gaussian Interaction Profile Kernel Similarity between $m_{i}$ and $m_{j}$, as expressed in the following formula: 


(6) \begin{align*}& \mathcal{MGIP}(m_{i}, m_{j})=exp\left( -\varphi_{m} \left \| BV(m_{i})-BV(m_{j}) \right \|^{2} \right)\end{align*}

Similarly, the spectral similarity of Gaussian interactions between diseases can be calculated as follows: 


(7) \begin{align*}& \mathcal{DGIP}(d_{i}, d_{j})=exp\left( -\varphi_{d} \left \| BV(d_{i})-BV(d_{j}) \right \|^{2} \right)\end{align*}

where the parameter $\varphi _{m}$, which primarily governs the bandwidth of the kernel, can be derived from the hyperparameter $\varphi _{m}^{^{\prime}}$ normalized by the average interaction count of each miRNA. Its computation can be accomplished through the following means: 


(8) \begin{align*} & \varphi_{m} =\frac{\varphi_{m}^{^{\prime}}}{\frac{1}{n_{m}} \sum_{i=1}^{n_{m}} \left \| BV(m_{i}) \right \|^{2}} \end{align*}

(9) \begin{align*} & \varphi_{d} =\frac{\varphi_{d}^{^{\prime}}}{\frac{1}{n_{d}} \sum_{i=1}^{n_{d}} \left \| BV(d_{i}) \right \|^{2} } \end{align*}

Based on prior research [32], both $\varphi _{m}^{^{\prime}}$ and $\varphi _{d}^{^{\prime}}$ here are set to 1. Here, $n_{m}$ and $n_{d}$, respectively, denote the number of miRNAs and diseases in the datasets.

### The feature matrices for miRNAs and diseases

As described in the previous sections, the feature matrix for miRNAs in this paper primarily relies on the functional similarity matrix. However, due to the limited number of miRNAs with functional similarity, the issue of matrix sparsity arises. To address this, Gaussian Interaction Profile Kernel Similarity for miRNAs was utilized for imputation. Similarly, the feature matrix for diseases was constructed based on semantic similarity, and Gaussian Interaction Profile Kernel Similarity for diseases was employed as imputation to alleviate the sparsity issue. Consequently, the feature matrix for miRNAs and diseases are as follows: 


(10) \begin{align*} & \mathcal{F}_{m}(m_{i}, m_{j}) \nonumber \\ &=\left\{\begin{matrix} \mathcal{FMRS }(m_{i}, m_{j}) & functional\, \;similarity\, \;between\, \;m_{i}\, \;and\, \; m_{j} \\ \mathcal{MGIP }(m_{i}, m_{j}) & otherwise \end{matrix}\right. \end{align*}

(11) \begin{align*} & \mathcal{F}_{d}(d_{i}, d_{j})=\left\{\begin{matrix} \mathcal{MDSS }(d_{i}, d_{j}) & semantic\, \;similarity\, \;between\, \;d_{i}\, \;and\, \; d_{j} \\ \mathcal{MGIP }(d_{i}, d_{j}) & otherwise \end{matrix}\right. \end{align*}

## HHOMR

**Figure 3:**
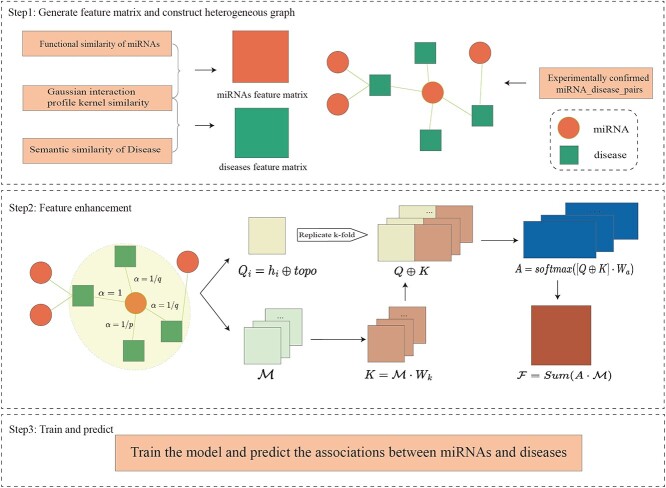
Overall framework of HHOMR. $\alpha $, $p$, and $q$ are correction coefficient, Return parameter and In-out parameter, respectively. ${Q}_{i}\in{\mathbb{R}}^{N\times ({D}_{feature} + {D}_{topo})}$ is the node’s initial embedding fused with $topo$ which is the topological embedding as the feature matrix of the graph. $\mathcal{M} \in{\mathbb{R}}^{k\times N\times ({D}_{feature} + {D}_{topo})}$ denotes the hybrid high-order moment embedding of the first-order neighbors centered on each node. ${Q} \oplus{K}$ denotes the fusion of the node embeddings with the hybrid high-order moment embeddings of the neighbors as an input to the calculation of element-level attention values. $A$ represents the element-level attention value of each moment. $W_{k}$ and $W_{a}$ are both learnable weight parameter matrices.

In this paper, we proposed a residual network model based on hybrid high-order moments for predicting associations between miRNAs and diseases, as shown in Fig. 3. The core idea behind this model is to utilize moments as a substitute for traditional aggregation methods(*e.g.* mean or sum) to capture comprehensive node information. HHOMR can be described as three steps: (1) construct feature matrices for miRNAs and diseases based on functional similarity of miRNAs, semantic similarity of diseases and Gaussian interaction profile kernel similarity; construct a heterogeneous graph of miRNAs and diseases based on validated MDAs; (2) fuse community and structural information in the heterogeneous graph, as well as hybrid high-order moments information and utilizing element-level attentions to enhance the node features; (3) train the whole model in an end-to-end way using a cross-entropy loss function and predict potential associations between miRNAs and diseases.

### Constructing a heterogeneous graph

Heterogeneous graph is a graph structure that includes two or more types of nodes and edges. The HHOMR model is based on this structure and incorporates two types of nodes: disease and miRNA. In order to map the HMDD2.0 datasets into the graph structure, we treated miRNAs and diseases as separate nodes, which were represented by the feature matrices $\mathcal{F}_{m}$ (495 dimensions) and $\mathcal{F}_{d}$ (383 dimensions), respectively. Next, to keep the dimensions of $\mathcal{F}_{m}$ and $\mathcal{F}_{d}$ consistent, we applied linear transformations to both, projecting them to 64 dimensions for processing. In addition, the adjacency matrix of heterogeneous graphs was mainly based on the association matrix $\mathcal{R}$. However, the data consisted of 189 585 elements and only 5430 elements were confirmed to be associated. To solve the problem of severe sparsity and positive and negative sample imbalance, we randomly selected 5430 elements from the remaining 184 155 elements as negative samples, and enhanced the asymmetric graph by adding edges in the forward and reverse, thus alleviating the above problems.

### Topological information

After constructing a heterogeneous graph about the association of miRNAs with diseases, we first considered the topological information of the graph as a whole. Based on previous research on network topology and the principle of network homogeneity, it is evident that within graph neural networks, the information pertaining to a node is not solely dependent on its inherent features. The topological structure information is also of importance. For nodes within the graph, their topological information can be broadly categorized into two types: community-related information and structural attributes. Therefore, we employed the node2vec algorithm [33] as a foundational method to capture this topological information. In the process of converting the graph structure into a sequence, random walks were employed to sample the target nodes. It should be noted that this random sampling is not entirely arbitrary. To ensure the comprehensive acquisition of both community and structural information, a biased sampling strategy was adopted as below: 


(12) \begin{align*}& \mathcal{P}(n_{i}=x\mid n_{i-1}=v)\left\{\begin{matrix} \alpha(v, x) & if\ (v, x)\in E\\ 0 & otherwise \end{matrix}\right.\end{align*}

where $\mathcal{P}$ is the probability of initiating from node $v$ and proceeding to node $x$ through a random walk. $E$ is the edge set of the graph. $\alpha $ is the concrete probability occurring when an edge is present between nodes $v$ and $x$. 


(13) \begin{align*}& \alpha(v, x)\left\{\begin{matrix} \dfrac{1}{p} & if\;d_{v, x}=0 \\ 1 & if\;d_{v, x}=1 \\ \dfrac{1}{q} &if\;d_{v, x}=2 \end{matrix}\right.\end{align*}

where the hyperparameters $p$ and $q$ serve as critical regulators, guiding the sampling process either deeper into the graph structure or in a broader manner. Specifically, when the hyperparameter $p$ is set to a lower value, the random walk sampling predominantly encompasses nodes that are immediate neighbors to the current node. Conversely, a lower value of $q$ propels the sampling process to favor nodes situated at a considerable distance from the current node. The parameter $d$ denotes the shortest path between nodes $v$ and $x$. Subsequent to the generation of a random walk sequence through sampling, the skip-gram algorithm [34] was employed to calculate topological structure embeddings of the nodes.

### High-order moments layer

In addition to topological information, we also enriched the feature information of the nodes themselves. Differentiating from other models, we considered higher order statistical information of the nodes. Traditional graph neural networks, in their feature processing step, often aggregate neighborhood information by means of either mean or max pooling to update the central node’s feature information. However, such aggregation methods overlook a plethora of node distribution information. From a statistical perspective, the statistical information of samples encompasses not only expectations but also higher-order central moments and higher-order origin moments. Indeed, many researchers [35–37] have recognized this and delved into the study of neighborhood distributions. Nevertheless, research on high-order mixed-domain information and its application in the prediction of associations between miRNAs and diseases remains scarce. Building upon their research, this paper integrates the richer and more complex distributional information from the domain to enhance the features of nodes.

To process the feature information of the entire graph, we first integrated the features of all nodes in the graph. Each node’s initial features consist of two parts: the first part is the node’s attribute feature matrix, and the second is the node’s topological information matrix. Since the attribute feature matrix dimensions for miRNAs and diseases are different, we employed a matrix transformation approach here to standardize their dimensions before integrating them with the topological information matrix. The specific projecting process is illustrated as follows: 


(14) \begin{align*}& \mathcal{F}_{all}=\begin{bmatrix} \mathcal{F}_{m}\cdot W_{m} \\ \mathcal{F}_{d}\cdot W_{d} \end{bmatrix}\parallel \mathcal{F}_{topo}\end{align*}

where $W_{m}$ and $W_{d}$ are both learnable linear transformation matrices. The role of $W_{m}$ is to project the 495-dimensional miRNAs feature matrix to 64 dimensions. And similarly, $W_{d}$ projects the 383-dimensional diseases feature matrix to 64 dimensions. Subsequently, the processed feature matrices as described above were used as inputs to calculate high-order moment features for each node. In statistics, the calculation of moments for a random variable is as follows: 


(15) \begin{align*}& A_{k}=\frac{1}{n}\sum_{i=1}^{n}X_{i}^{k}\end{align*}

From the above formula, it can be observed that when $k$ is equal to 1, the calculation yields the sample’s expectation, which corresponds to the commonly used average aggregation method in traditional GCN. However, since we intended to apply this to a graph-structured model and kept the order of $k$-moments consistent, we needed to make some adjustments to the formula. This is shown as follows: 


(16) \begin{align*}& \mathcal{M}_{k}^{l+1}(v_{i})=\left(\frac{1}{n}\sum_{j\in N(v_{i})}\left(h_{j}^{l}\right)^{k}\right)^{\frac{1}{k}}\cdot W_{k}\end{align*}

where $N(v_{i})$ represents the set of neighboring nodes of node $v_{i}$, where $n$ denotes the number of neighbors of $v_{i}$, $k$ represents the order of the moment, $W_{k}$ is a learnable parameter matrix, and $h_{j}^{l}$ stands for the feature vector of node $j$ in the $l$th layer. The role of $\frac{1}{k}$ here is to normalize the $k$th order moment feature vector.

In addition to the origin moments, we also took into account the $k$th central moments as alternative information, which will be empirically tested for its impact on the model’s performance in subsequent experiments. The specific calculation process for $k$th central moments is as follows: 


(17) \begin{align*}& \mathcal{M}_{k}^{l+1}(v_{i})=\left(\frac{1}{n}\sum_{j\in N(v_{i})}\left(h_{j}^{l}-\frac{1}{n}\sum_{j\in N(v_{i})}h_{j}^{l}\right)^{k}\right)^{\frac{1}{k}}\cdot W_{k}\end{align*}

where the roles of all variables in the equation are similar to those in the calculation of $k$th origin moments.

### Attention layer

After computation, we obtained high-order moment features for each node. Instead of simple and indiscriminate average fusion, we assigned a learnable weight parameter to each order moment for each node, allowing the model to adaptively select the most important moment information. Hence, we introduced an attention mechanism. Specifically, we used the feature vectors processed in the previous layer and the multi-order moments corresponding to each node as inputs to the attention layer. While utilizing moment information, we also retained and learned from the hidden layer’s information. The attention score calculation for the $k$-order moment of node $v_{i}$ in layer $l+1$ is formulated as follows: 


(18) \begin{align*}& \alpha_{k}^{l+1}(v_{i})=sigmoid\left([h_{i}^{l}\cdot W_{q}^{l+1}\parallel \mathcal{M}_{k}^{l+1}(v_{i})\cdot W_{k}^{l+1}]\cdot W_{\alpha }^{l+1}\right)\end{align*}

where $W_{q}$, $W_{k}$, and $W_{\alpha }$ are all learnable matrices. $h_{i}^{l}$ represents the embedding of the $i$th node in layer $l$, and $\mathcal{M}_{k}^{l+1}(v_{i})$ is the embedding of the $k$-order moment of node $v_{i}$ in layer $l$. Then, the weight parameters $\alpha $ calculated above are multiplied by their corresponding moment embeddings and summed. This results in the embedding of node $i$ in layer $l$, and the calculation formula is as follows: 


(19) \begin{align*}& h_{i}^{l+1}=\sum_{k=1}^{K}\alpha_{k}^{l+1}(v_{i})\odot \mathcal{M}_{k}^{l+1}(v_{i})\end{align*}

where the symbol $\odot $ signifies the component-wise multiplication of the weight parameter $\alpha $ with its corresponding moment embedding. Through the aforementioned process, the distributional information of each node within the graph can be effectively integrated, resulting in the culmination of the final node embeddings.

### Residual layer

After the structural information of the heterogeneous graph as well as the information of the nodes themselves are fused, the model’s work of feature enhancement for the data is basically completed. However, the overall architecture of the model still has some shortcomings. We have observed during experimentation that gradient vanishing can occur in certain scenarios. This phenomenon is primarily attributed to the increasing model depth. Furthermore, our datasets are relatively small, making it susceptible to uneven data distribution, resulting in occasional variability in experimental outcomes.

To address these issues, we implemented the following strategies. First, to tackle the challenge posed by the small datasets, we employed a five-fold cross-validation approach, utilizing each portion of the data as a validation set in turn to maximize data utilization. Additionally, we introduced residual structures. These structures enabled us to preserve the original node features while processing the features derived from nodes, thereby mitigating the issue of gradient vanishing. The computational formula for the residual structure is presented below: 


(20) \begin{align*}& \mathcal{F}_{u}=\beta *\mathcal{F}_{o}\oplus \gamma *\mathcal{F}_{s}\end{align*}

where the hyperparameters $\gamma $ and $\beta $ play a pivotal role in regulating the ratio between node features before and after enhancement. $\mathcal{F}_{s}$ represents the feature matrix before processing, while $\mathcal{F}_{o}$ denotes the feature matrix after processing.

## Result

### Experiment settings and performance evaluation

**Figure 4:**
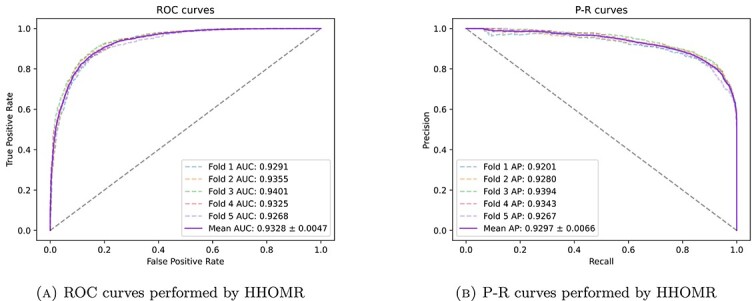
Five-fold cross-validation of HHOMR.

The training datasets utilized in this study comprises data from HDMMv2.0, encompassing 495 miRNA types and 383 disease types, with a total of 5430 known associations existing between these two major categories of data. The model proposed in this paper is primarily implemented using PyTorch and a graph-based framework. Consequently, we structured this data into a graph format and generated an adjacency matrix. It is evident that, in comparison to the known association data, the scale of unknown information is relatively small. If we were to merely categorize the unknown associations as irrelevant, a significant class imbalance issue between positive and negative samples would manifest. To address this concern, we employed the quantity of known association data as a benchmark and randomly selected 5430 unknown associations from the remaining data, classifying them as negative samples. Importantly, these negative samples do not necessarily imply the absence of an association. On the contrary, they represented instances where association information was unclear. This approach effectively rectified the class imbalance problem between positive and negative samples. Furthermore, given the modest overall datasets size, potential experimental result fluctuations needed to be addressed. To mitigate this, we employed a five-fold cross-validation strategy to assess the model’s performance. This strategy involved partitioning the data into five equal subsets, employing four subsets for training in each iteration, and reserving one subset for testing. The final results were obtained by averaging the outcomes of these five experiments. Figure 4 illustrates the results of the five-fold cross-validation, yielding AUC scores of 0.9291, 0.9355, 0.9401, 0.9325, and 0.9268 in each fold. The average AUC score across these folds amounts to 0.9328. It is discernible that variations in data distribution have a certain impact on the model, but the model’s performance remains relatively stable.

### Hyperparameter experiment

**Figure 5:**
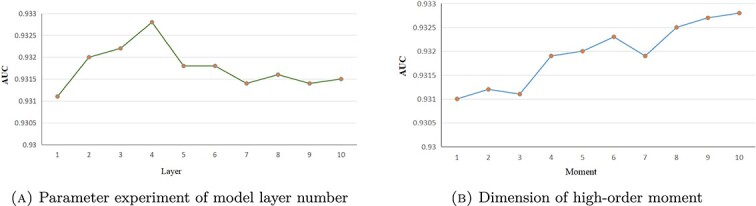
Results of employing different layer and moment values in HHOMR.

Our proposed model is influenced by several hyperparameters, the more critical of which are the number of model layers and the order of moments. Here, we experimented with different parameter values to assess the performance of HHOMR. First, we examined the model layers. The initial value for the ’layer’ parameter is set to 1, with a step size of 1 for 10 experiments. The results, as depicted in Fig. 5(a), show that the model performs optimally when ’layer’ is set to 4. This observation is attributed to the fact that a lower number of layers results in less aggregated information, which adversely affects model performance. Conversely, higher layer numbers lead to increased computational complexity and potential convergence issues. Subsequently, while keeping the other parameters at their optimal values, we experimented with the ’moment’ parameter. Initially set to 1, we conducted 10 experiments with a step size of 1. As illustrated in Fig. 5(b), the model achieves its best performance when ’moment’ is set to 10. This outcome aligns with our previous description, highlighting that richer distributional information enhances model performance. Furthermore, we explored an additional possibility of enriching the model by incorporating not only the origin moments but also the central moments information. Interestingly, we found that, for the HMDD2.0 datasets, the model performs best when utilizing only origin moments.

### Comparison with baseline

**Figure 6:**
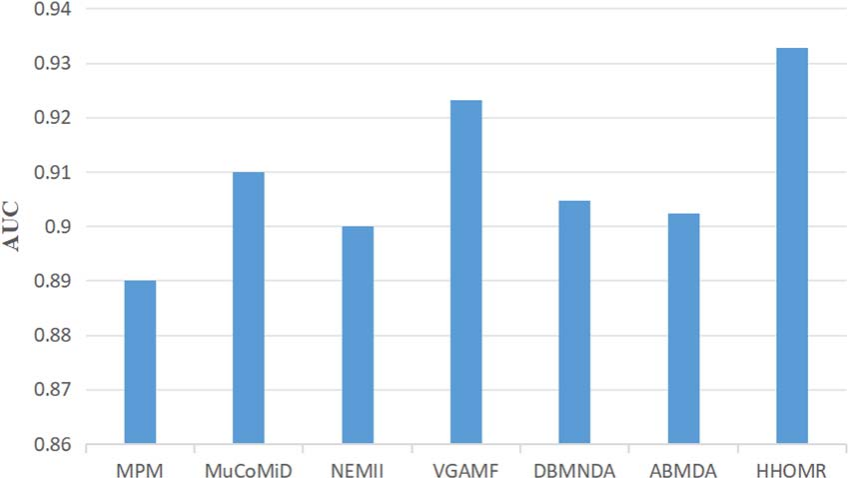
Performance comparison between HHOMR and other six methods in terms of AUC on five-fold cross-validation.

To further demonstrate the superiority of HHOMR in identifying miRNA–disease associations, we compared it with several state-of-the-art models. The first one is the multitasking graph convolution based approach MuCoMiD [38], a model that allows for automated feature extraction characterization while integrating knowledge from five heterogeneous bioinformatic sources into a multitasking environment. The second model is the network embedding-based multi-information integration approach NEMII [39], which is modeled as a semi-supervised deep model of multilayer nonlinear functions, structured deep network embedding approach (SDNE) as the backbone, and random forests are used as a classification engine to classify miRNA–disease pairs. The third model is the MPM [40] which utilizes information from multiple data sources to enhance features through an information transfer mechanism, the main idea of this model is to train the SDNE by enriching the known miRNA/disease-protein-coding gene (PCG) association information and augmenting the features to ultimately predict the association probability. The fourth model is VGAMDF [41] using a variant autoencoder, which combines disease similarity and miRNA similarity to construct two spliced matrices, respectively, to train the autoencoder to finally output the association score. In addition, we also compared the adaptive enhanced miRNA disease association prediction model (ABMDA) [42] and deep-belief network for miRNA–disease association prediction (DBNMDA) [43] in terms of AUC metrics alone to fully demonstrate the superiority of our model performance. These methods have been proposed in recent years to ensure a more practical and relevant comparative experiment. To maintain consistency between the data generated by the comparative experiment’s models and the original results, we also employed five-fold cross-validation and compared the results based on the average outcomes, as presented in Table 1, Fig. 6. It is noteworthy that our proposed model outperforms the aforementioned models significantly. This can primarily be attributed to our model’s consideration of each node’s distribution characteristics during feature processing, as opposed to solely focusing on expectations. Additionally, structural feature orders are also encompassed, contributing to the model’s commendable performance.

**Table 1:** The performance of five methods on miRNA–disease association prediction

Model	AUC.(%)	Acc.(%)	Pre.(%)	F1.(%)
MPM	89.11	81.30	81.00	81.10
MuCoMiD	91.21	**90.00**	82.80	82.80
NEMII	90.60	81.50	81.40	81.40
VGAMF	92.33	84.91	84.83	85.27
HHOMR	**93.28**	86.25	**85.54**	**85.65**

### Case studies

To further enhance the persuasiveness of our results, we performed case studies on esophageal neoplasms, lymphoma, and prostate neoplasms. Specifically, first we slightly adjusted the construction of the training and test sets. We labeled the edges of the miRNA–disease heterogeneity graph, which connect specific diseases and miRNAs, as the test set, and labeled all other edges as the training set to train the HHOMR. We then used the trained model to generate correlation scores between specific diseases and miRNAs. We ranked the predicted association scores from high to low, with the highest ranked miRNAs having a high likelihood of being associated with the disease. Finally, based on the association scores, the dbDEMC [44] and miR2disease [10] datasets were used to identify associations between specific diseases and miRNAs. The miR2Disease and dbDEMC datasets contain 697 and 1816 known MDA pairs respectively, encompassing 14 diseases. In the case studies for prostate neoplasms, lymphoma, and esophageal neoplasms, the datasets include 203, 193, and 279 known MDA pairs, respectively.

The initial selection focused on prostate tumors as the experimental subject. Over half of males encounter prostate-related conditions in their lifetime, and without proper treatment, these conditions may potentially progress to cancer. Thus, early-stage treatment becomes crucial. MiRNA plays a pivotal role in the occurrence, development, and prognosis assessment of prostate cancer. Investigating the correlation between prostate tumors and miRNAs can aid in understanding their etiology and discovering diagnostic biomarkers. As depicted in Table 2, among miRNAs associated with prostate tumors, 46 out of the top 50 can be verified through the dbDEMC and miR2Disease datasets. Although mir-125b was not verified in these two databases, some studies have shown that elevated levels of mir-125b have been detected in prostate cancer, and experts believe that mir-125b can down-regulate anti-apoptotic proteins, which leads to a decrease in apoptosis and an increase in cell proliferation, and thus promotes the growth of prostate tumors [45].

**Table 2:** Top 50 miRNAs related to prostate neoplasms predicted by HHOMR

miRNA (1-25)	Evidence	miRNA (26-50)	Evidence
hsa-mir-221	dbDEMC and miR2Disease	hsa-let-7i	dbDEMC
hsa-mir-125b	Unconfirmed	hsa-mir-15b	dbDEMC
hsa-mir-34a	dbDEMC	hsa-mir-34c	Unconfirmed
hsa-mir-223	dbDEMC	hsa-let-7f	dbDEMC
hsa-mir-222	dbDEMC	hsa-mir-214	dbDEMC
hsa-mir-145	dbDEMC and miR2Disease	hsa-let-7g	dbDEMC
hsa-let-7a	dbDEMC	hsa-mir-141	dbDEMC
hsa-mir-29b	dbDEMC	hsa-mir-106a	dbDEMC and miR2Disease
hsa-mir-29a	dbDEMC	hsa-mir-34b	dbDEMC
hsa-mir-1	dbDEMC	hsa-mir-7	dbDEMC
hsa-let-7b	dbDEMC	hsa-mir-148a	dbDEMC
hsa-mir-143	dbDEMC and miR2Disease	hsa-mir-206	dbDEMC
hsa-let-7c	dbDEMC	hsa-mir-182	dbDEMC
hsa-mir-199a	dbDEMC	hsa-mir-27a	dbDEMC
hsa-mir-146b	Unconfirmed	hsa-mir-196a	dbDEMC
hsa-mir-106b	dbDEMC	hsa-mir-183	dbDEMC
hsa-mir-31	dbDEMC	hsa-mir-30a	dbDEMC
hsa-let-7e	dbDEMC and miR2Disease	hsa-mir-100	dbDEMC
hsa-mir-9	dbDEMC	hsa-mir-93	dbDEMC
hsa-mir-195	dbDEMC	hsa-mir-205	dbDEMC
hsa-mir-181b	dbDEMC	hsa-mir-107	dbDEMC
hsa-let-7d	dbDEMC	hsa-mir-10b	dbDEMC
hsa-mir-142	Unconfirmed	hsa-mir-22	dbDEMC
hsa-mir-133a	dbDEMC	hsa-mir-132	dbDEMC
hsa-mir-25	dbDEMC	hsa-mir-23b	dbDEMC

Additionally, esophageal tumors were subjected to correlation experiments. Given their relatively high incidence and mortality rates, it is of paramount importance to predict their trends in early-stage disease development. The association prediction between esophageal tumors and miRNAs holds significant relevance. Table 3 reveals that out of the top 50 miRNAs associated with esophageal tumors, 46 can be confirmed using the dbDEMC and miR2Disease datasets. For example, mir-155 is involved in complex interactions within the tumor microenvironment, especially with cancer-associated fibroblasts (CAF). CAF affected by mir-155 can influence tumor progression, metastasis and drug resistance. In addition, mir-155 can be transferred between different cell types via exosomes, affecting a variety of processes such as tumor cell migration, invasion, and the development of a more aggressive cancer phenotype [46].

**Table 3:** Top 50 miRNAs related to esophageal neoplasms predicted by HHOMR

miRNA (1-25)	Evidence	miRNA (26-50)	Evidence
hsa-mir-155	dbDEMC	hsa-mir-143	dbDEMC and miR2Disease
hsa-mir-17	miR2Disease	hsa-let-7e	dbDEMC
hsa-mir-21	dbDEMC and miR2Disease	hsa-mir-24	dbDEMC and miR2Disease
hsa-mir-20a	miR2Disease	hsa-let-7c	dbDEMC and miR2Disease
hsa-mir-16	dbDEMC and miR2Disease	hsa-let-7b	dbDEMC and miR2Disease
hsa-mir-19b	dbDEMC and miR2Disease	hsa-mir-200c	dbDEMC
hsa-mir-92a	Unconfirmed	hsa-mir-210	miR2Disease
hsa-mir-146a	miR2Disease	hsa-mir-199a	dbDEMC and miR2Disease
hsa-mir-18a	Unconfirmed	hsa-mir-200b	Unconfirmed
hsa-mir-221	dbDEMC and miR2Disease	hsa-mir-146b	Unconfirmed
hsa-mir-126	dbDEMC and miR2Disease	hsa-mir-133a	dbDEMC
hsa-mir-19a	dbDEMC	hsa-mir-106b	dbDEMC
hsa-mir-223	dbDEMC and miR2Disease	hsa-mir-133b	dbDEMC
hsa-mir-34a	dbDEMC and miR2Disease	hsa-mir-31	dbDEMC and miR2Disease
hsa-mir-150	dbDEMC	hsa-mir-181b	dbDEMC and miR2Disease
hsa-mir-15a	dbDEMC and miR2Disease	hsa-let-7d	dbDEMC and miR2Disease
hsa-mir-29a	dbDEMC and miR2Disease	hsa-mir-30a	miR2Disease
hsa-mir-29b	dbDEMC and miR2Disease	hsa-mir-9	dbDEMC
hsa-mir-222	dbDEMC and miR2Disease	hsa-let-7f	dbDEMC and miR2Disease
hsa-mir-195	dbDEMC and miR2Disease	hsa-mir-148a	miR2Disease
hsa-let-7a	dbDEMC and miR2Disease	hsa-mir-15b	dbDEMC
hsa-mir-1	dbDEMC	hsa-let-7i	dbDEMC
hsa-mir-181a	dbDEMC and miR2Disease	hsa-mir-34c	dbDEMC
hsa-mir-29c	dbDEMC	hsa-mir-26a	dbDEMC and miR2Disease
hsa-mir-142	Unconfirmed	hsa-mir-125a	dbDEMC and miR2Disease

Lastly, lymphatic tumors were chosen as the experimental focus. Lymphatic tumors encompass cancers involving the lymphatic system, including Hodgkin’s lymphoma and non-Hodgkin’s lymphoma. These diseases exhibit substantial clinical complexity, requiring diverse treatment strategies. Therefore, it is essential to infer the development of different types of lymphatic tumors based on the aberrant expression of various miRNAs. As indicated in Table 4, among miRNAs related to lymphatic tumors, 47 out of the top 50 can be validated using the dbDEMC and miR2Disease datasets. Studies on the role of mir-17, mir-19b, and mir-18a in lymphoid neoplasms, particularly chronic lymphocytic leukemia, have shown that these miRNAs are potential cancer biomarkers, and their aberrant expression all signify lymphoid tumorigenesis [47, 48].

**Table 4:** Top 50 miRNAs related to lymphoma predicted by HHOMR

miRNA (1-25)	Evidence	miRNA (26-50)	Evidence
hsa-mir-17	dbDEMC	hsa-mir-125a	dbDEMC
hsa-mir-19b	dbDEMC	hsa-mir-30a	dbDEMC
hsa-mir-18a	dbDEMC	hsa-mir-106a	dbDEMC
hsa-mir-221	dbDEMC	hsa-mir-133b	dbDEMC
hsa-mir-16	dbDEMC	hsa-mir-7	dbDEMC
hsa-mir-29b	dbDEMC	hsa-mir-182	dbDEMC
hsa-mir-29a	dbDEMC	hsa-mir-206	dbDEMC
hsa-mir-125b	dbDEMC	hsa-mir-122	Unconfirmed
hsa-mir-222	dbDEMC	hsa-mir-107	dbDEMC and miR2Disease
hsa-let-7e	dbDEMC	hsa-mir-10b	dbDEMC
hsa-mir-1	dbDEMC	hsa-mir-132	dbDEMC
hsa-mir-195	dbDEMC	hsa-mir-199b	dbDEMC
hsa-mir-24	dbDEMC	hsa-mir-93	dbDEMC
hsa-let-7d	dbDEMC	hsa-mir-20b	dbDEMC
hsa-mir-146b	dbDEMC	hsa-mir-124	dbDEMC
hsa-mir-200b	dbDEMC	hsa-mir-30c	dbDEMC
hsa-mir-181a	dbDEMC	hsa-mir-23b	dbDEMC
hsa-mir-106b	dbDEMC	hsa-mir-218	Unconfirmed
hsa-let-7i	dbDEMC	hsa-mir-18b	dbDEMC
hsa-let-7f	Unconfirmed	hsa-mir-27b	dbDEMC
hsa-mir-9	dbDEMC	hsa-mir-429	dbDEMC
hsa-mir-142	dbDEMC	hsa-mir-127	dbDEMC
hsa-let-7g	dbDEMC	hsa-mir-23a	dbDEMC
hsa-mir-181b	dbDEMC	hsa-mir-224	dbDEMC
hsa-mir-15b	dbDEMC	hsa-mir-335	dbDEMC

## Conclusion

By investigating the relationship between miRNAs and diseases, complicated disease early detection and diagnosis may be assessed. Therefore, in order to forecast the potential correlation between miRNAs and diseases, we suggest using the hybrid high-order moment model. In particular, the performance of HHOMR is influenced by three key aspects. (1) The topological layer uses node2vec algorithm to extract the structural features of the graph, so that nodes can pay attention to the global information; (2) the hybrid high-order moment encoder layer aggregates more distribution information of nodes to ensure the integrity of information; (3) the element-level attention layer weights the calculated high-order moments so that each feature pays more attention to the distribution information with the greatest influence. In order to demonstrate the effectiveness of our proposed model, in the experimental stage, we adopted the method of five-fold cross-validation and studied three classic cases such as lymphatic tumors. Experiments show that HHOMR fully demonstrates the superiority of the model. In the future, our work will concentrate on enhancing the accuracy of predictions. This focus is critical in the medical industry, where every increment in accuracy can significantly enhance patient outcomes and provide greater assurance for patient care.

**Key Points**

- The occurrence of diseases is closely related to the abnormal expression of miRNAs.
- The traditional wet experiments are time-consuming and costly, while the calculation method greatly improves the efficiency of the traditional method.
- In this paper, we propose a residual model (HHOMR) based on hybrid high-order moments and element-level attentions to predict the associations between miRNAs and diseases.
- Through comparative experiments, case studies, and five-fold cross-validation, HHOMR has demonstrated considerable potential in predicting the associations between miRNAs and diseases.

## Data Availability

All data and codes can be available at https://github.com/W-LP/HHOMR.
