# Correction to: HHOMR: a hybrid high-order moment residual model for miRNA-disease association prediction

**DOI:** 10.1093/bib/bbae684

**Published:** 2024-12-17

This is a correction to: Zhengwei Li, Lipeng Wan, Lei Wang, Wenjing Wang, Ru Nie, HHOMR: a hybrid high-order moment residual model for miRNA-disease association prediction, *Briefings in Bioinformatics*, Volume 25, Issue 5, September 2024, bbae412, https://doi.org/10.1093/bib/bbae412

In the originally published version of the manuscript, there was erroneous mark-up for the first corresponding author in the byline. This should rbe: ``Lei Wang'' instead of: ``Wenjing Wang''. Emendation has been made in the article.

